# Interleukin‐2 promotes pegylated interferon alpha for hepatitis B surface antigen loss: A retrospective pragmatic clinical study at the Fourth Affiliated Hospital of Zhejiang University Medical College

**DOI:** 10.1002/hsr2.932

**Published:** 2022-11-14

**Authors:** Wencai Qi, Yuming Wang, Guangyu Huang, Kaifa Wang

**Affiliations:** ^1^ School of Mathematics and Statistics Southwest University Chongqing People's Republic of China; ^2^ Institute for Infectious Diseases, Southwest Hospital Army Medical University Chongqing People's Republic of China; ^3^ Public Health Hospital of Southwest University Chongqing People's Republic of China; ^4^ Department of Infectious Diseases The Fourth Affiliated Hospital Zhejiang University School of Medicine Yiwu Zhejiang People's Republic of China

**Keywords:** hepatitis B surface antigen, interleukin‐2, intervention benefit, kinetic pattern, pegylated interferon alpha

## Abstract

**Background and Aims:**

Interleukin‐2 (IL‐2) can be used as an adjuvant therapy when pegylated interferon alpha (Peg‐IFN‐α) does not effectively promote hepatitis B surface antigen (HBsAg) loss, but the relevant timing, kinetic patterns, and prognostic associations of this intervention are unclear.

**Methods:**

A total of 115 patients with chronic hepatitis B (CHB) treated at our institution between October 2018 and March 2021 were included in this retrospective analysis. They were divided into two kinetic patterns by using K‐medoids cluster analysis. Profile and prognostic associations were statistically analyzed between the two patterns.

**Results:**

After baseline standardization, before the intervention, the relative HBsAg level showed a continuously increasing trend, but after the intervention, it showed a continuously decreasing trend. Based on the relative change in the HBsAg level, two kinetic patterns, namely, a fluctuation platform pattern and a stepwise growth pattern, were identified by using K‐medoids cluster analysis for all 115 patients before IL‐2 intervention. Profile analysis showed that there were statistically significant differences between the two patterns before IL‐2 intervention (*p* < 0.05), but their profiles showed the same trend after 2 weeks of IL‐2 intervention. Prognostic association analysis showed that CD8+ T cells, alanine transaminase (ALT), age, natural killer (NK) cells, neutrophils, and course of treatment before IL‐2 intervention were the six main indicators affecting the relative decrease in the HBsAg level.

**Conclusion:**

For CHB patients who have received continuous Peg‐IFN‐α treatment, IL‐2 intervention should be given as early as possible when the HBsAg level has not decreased for four consecutive weeks or a fluctuation platform pattern is observed. After the intervention, a downward relative change in the HBsAg level can be maintained over 4 weeks. CD8+ T cells, ALT, NK cells, and neutrophils are baseline indicators closely related to the prognosis of this intervention.

## 1 INTRODUCTION

Hepatitis B virus (HBV) infection leads to chronic hepatitis B (CHB) in hundreds of millions of people and is one of the most common causes of cirrhosis, hepatocellular carcinoma, and viral hepatitis‐related death; thus, HBV infections are a major global health problem.^1^ Pegylated interferon alpha (Peg‐IFN‐α) is one of the most commonly used antiviral drugs for chronic HBV infection. Interferon can activate the immune system to eliminate HBV, and compared with nucleos(t)ide analogues (NAs) as antiviral drugs, interferon may more effectively reduce the overall incidence rate of cirrhosis/liver cancer in HBV‐infected patients^2^; nevertheless, clinically, there are significant individual differences in the response to interferon therapy.^3^ For instance, at different stages of treatment, the poor response may be related to the depletion of immune function and the number and function of cytotoxic T lymphocytes or natural killer (NK) cells.^4^ In clinical practice, interleukin‐2 (IL‐2), granulocyte‐macrophage stimulating factor, and therapeutic vaccines have been used to improve efficacy,^5^ but the relevant timing, kinetic patterns, and prognostic associations of these interventions are unclear.^4^

As an important nonspecific immune enhancer, IL‐2 can enhance the activity and number of NK cells.^6^, ^7^, ^8^ In addition, an appropriately low dose of IL‐2 can enhance regulatory T cells (Tregs), and strengthen the control and regulation of proinflammatory NK cells^9^ and thus, promote the efficiency of interferon in hepatitis B surface antigen (HBsAg) loss. Injection of IL‐2 once a week will not cause serious adverse reactions to the human body, and the common adverse reactions are transient redness, swelling, heat, and pain at the injection site. Based on clinical retrospective cases, the main objective of this paper is to evaluate the timing of IL‐2 intervention and the HBsAg loss efficiency after intervention and to preliminarily analyze the clinical benefits after IL‐2 intervention.

## 2 MATERIALS AND METHODS

### 2.1 Study population

A retrospective pragmatic clinical study was conducted using the case database of patient medical records. This study got an exemption from the Ethical Review Committee of the Fourth Affiliated Hospital Zhejiang University School of Medicine and the requirement for written informed consent was waived due to the retrospective nature of the study. From the hospital medical record database, clinical data for patients with CHB treated between October 2018 and March 2021 were extracted for retrospective analysis. All patients were from Zhejiang Province, China. The genetic makeup and dietary pattern of the patients were similar. The inclusion criteria were as follows: (1) aged between 18 and 65 years; (2) HBV infection for more than 6 months; (3) no contraindications of Peg‐IFN‐α therapy; (4) use of IL‐2 intervention during the treatment; and (5) Peg‐IFN‐α treatment performed for at least 4 weeks before IL‐2 intervention. The exclusion criteria were as follows: (1) patients with chronic hepatitis C and/or HIV coinfection; (2) patients with end‐stage liver diseases, such as decompensated cirrhosis and liver cancer; (3) patients with poor compliance with an interval between two visits of more than 4 weeks; and (4) patients who completed only one dose of IL‐2 intervention and had no follow‐up medical data. In this study, the time of IL‐2 injection was selected as the baseline time.

### 2.2 Data collection

Apart from the demographic indices, clinical information such as whether to conduct NAs treatment before the first visit to our institution, the levels of HBsAg, hepatitis B surface antibody (HBsAb), hepatitis B e‐antigen (HBeAg), HBV DNA, alanine transaminase (ALT), CD8+ T cells, NK cells, and neutrophils and the follow‐up time for diagnosis and treatment were also collected. The HBsAg/HBsAb/HBeAg kit from Abbott was used to identify and measure HBV serum markers (HBsAg, anti‐HBs, and HBeAg) by electrochemiluminescence immunoassay. Fluorescence quantitative polymerase chain reaction was utilized to detect the HBV DNA load (high sensitivity of immunomagnetic beads from Fosun Pharma) with a detection limit of 100 IU/ml. CD8+ T cell and NK cell counts were determined with a Gallios flow cytometer (Beckman‐Coulter Gallios), serum ALT levels were determined by colorimetry (Beckman‐Coulter AU5800), and the absolute neutrophil count was determined by flow cytometry (UniCel®DxH 800 Coulter).

### 2.3 Criteria for evaluation

If the HBV DNA load was greater than the detection limit, the diagnosis was viremia; otherwise, the diagnosis was aviremia. If the HBeAg level was greater than 1, the patient was diagnosed as HBeAg positive; otherwise, the patient was diagnosed as HBeAg negative. The immune‐active stage was diagnosed in patients with viremia if the ALT ≥30 IU/ml for males or ≥19 IU/ml for females; otherwise, the immune‐inactive stage was diagnosed.^10^, ^11^ Neutrophil levels were used to evaluate the severity of adverse reactions, that is, the lower the level was, the more serious the adverse reactions.

### 2.4 Statistical analysis

Categorical data were expressed as frequencies or percentages as needed, and the *χ* ^2^ test was performed. If the total sample size was less than 40 or the theoretical frequency was less than 5, Fisher's exact test was performed. The Shapiro−Wilk test was used to assess the normal distribution of quantitative data. Quantitative data subject to a normal distribution are expressed as the mean ± standard deviation; otherwise, they are expressed as the median with a corresponding range.

The level of HBsAg was measured once a week. Due to the difference in compliance, the data were missing in some weeks for a small number of patients. In this study, the missing data were filled by the average values of two adjacent measurements. To eliminate the magnitude difference in the level of HBsAg, (HBsAg at *i* ^th^ week/baseline HBsAg) × 100% was calculated to express the HBsAg level at every point in time, which was named the relative HBsAg level (if the value was larger than 100, the HBsAg level in the current week was higher than the baseline), and nonparametric Friedman rank test was performed to test the difference in the relative HBsAg levels at different time points.

Clinical studies have found that the level of HBsAg is closely related to the risk of cirrhosis and hepatocellular carcinoma.^12^ To characterize the change in the HBsAg level in the adjacent 2 weeks and eliminate the impact of magnitude differences, ([HBsAg at *i* ^th^ week – HBsAg at [*i*−1]^th^ week]/baseline HBsAg) × 100% was calculated to express the change in the level of HBsAg at every time point after the standardized baseline, which was named the relative change in the HBsAg level (if the value was positive, the level of HBsAg increased, i.e., the Peg‐IFN response was not good). Subsequently, two categories of the relative change in the HBsAg level were identified by using K‐medoids cluster analysis.^13^ The characteristics of the profile kinetic patterns were compared among different clusters by profile analysis.^14^ The comparison of baseline characteristics between the two categories of patients was performed using the independent sample *t*‐test, Mann−Whitney *U* test, and *χ* ^2^ test as needed. For the prognostic association analysis after IL‐2 intervention, stepwise regression analysis (backward) was performed.

Excel 2017 and MATLAB R2018a were used for data processing, and statistical tests were performed by using R (version 4.1.2) and IBM SPSS Statistics (version 22). For the two‐sided tests, a *p* < 0.05 was considered the significance threshold. Statistical explanations are provided in the supporting information.

## 3 RESULTS

### 3.1 Demographics and baseline characteristics

A total of 115 CHB patients met the inclusion/exclusion criteria and were statistically analyzed. Among all patients, 74.78% (86/115) were male, and 25.22% (29/115) were female. The patient demographics and baseline characteristics and the comparison of each variable between sexes are shown in Table 1.

**Table 1:** Demographics and baseline characteristics of all patients and comparison of each variable by gender

Variable	Value	Gender		Statistics	*p* Value
		Male (*n* = 86)	Female (*n* = 29)		
Age^a^ (years, mean ± SD, *n* = 115)	41.99 ± 10.55	42.48 ± 10.42	40.55 ± 11.01	*t* = 0.848	0.40
HBsAg^b^ (IU/ml, median [range], *n* = 115)	1574.41 (0.85−53,110.60)	1008.64 (0.85−53,110.60)	2181.78 (2.13−15,187.00)	*Z* = 0.747	0.46
Neutrophil level^a^ (10^9^/L, mean ± SD, *n* = 109)	1.53 ± 0.78	1.56 ± 0.79 (*n* = 83)	1.44 ± 0.75 (*n* = 26)	*t* = 0.658	0.51
Neutrophil level^a^ (10^9^/L, mean ± SD, *n* = 109)	1.53 ± 0.78	1.56 ± 0.79 (*n* = 83)	1.44 ± 0.75 (*n* = 26)	*t* = 0.658	0.51
Course of treatment before IL‐2 intervention^b^ (weeks, median [range], *n* = 115)	14 (5−125)	14 (5−125)	15 (6−53)	*Z* = 0.635	0.53
CD8^+^ T‐cell level^b^ (M/L, median [range], *n* = 49)	335 (81−1221)	335 (81−1221) (*n* = 37)	319.50 (82−937) (*n* = 12)	*Z* = 0.093	0.93
NK‐cell level^b^ (M/L, median [range], *n* = 48)	199 (6−1009)	199(41−1009) (*n* = 36)	200 (6−477) (*n* = 12)	*Z* = 0.310	0.76
NAs treatment before the first visit^c^ (cases [%], *n* = 115)				*χ* ^2^ = 0.082	0.77
Treated with NAs	70 (60.87%)	53 (75.71%)	17 (24.29%)		
No NAs treatment	45 (39.13%)	33 (73.33%)	12 (26.67%)		
HBeAg^c^ ^,^ ^d^ (cases [%], *n* = 37)				*χ* ^2^ = 4.405	0.10
Positive	35 (94.59%)	25 (71.43%)	10 (28.57%)		
Negative	2 (5.41%)	0 (0.00%)	2 (100.00%)		
HBV DNA^c^ ^,^ ^d^ (cases [%], *n* = 49)				*χ* ^2^ = 0.576	0.52
Aviremia	32 (65.31%)	24 (75.00%)	8 (25.00%)		
Viremia	17 (34.69%)	11 (64.71%)	6 (35.29%)		
Immune stage^c^ ^,^ ^d^ (cases [%], *n* = 45)				*χ* ^2^ = 2.045	0.17
Immune‐active stage	15 (33.33%)	9 (60.00%)	6 (40.00%)		
Immune‐inactive stage	30 (66.67%)	24 (80.00%)	6 (20.00%)		

Abbreviations: HBeAg, hepatitis B e‐antigen; HBsAg, hepatitis B surface antigen; HBV, hepatitis B virus; IL‐2, interleukin‐2; NAs, nucleos(t)ide analogues; NK, natural killer; SD, standard deviation.

^a^ Independent sample *t*‐test.

^b^ Mann−Whitney *U* test.

^c^ The *χ* ^2^ test and Fisher's exact test were performed if the theoretical frequency was less than 5.

^d^ Indicates that there are missing data for this index, and *n* in the corresponding parentheses represents the actual sample size.

### 3.2 Change in the relative level of HBsAg for 4 consecutive weeks before and after IL‐2 intervention

**Figure 1:**
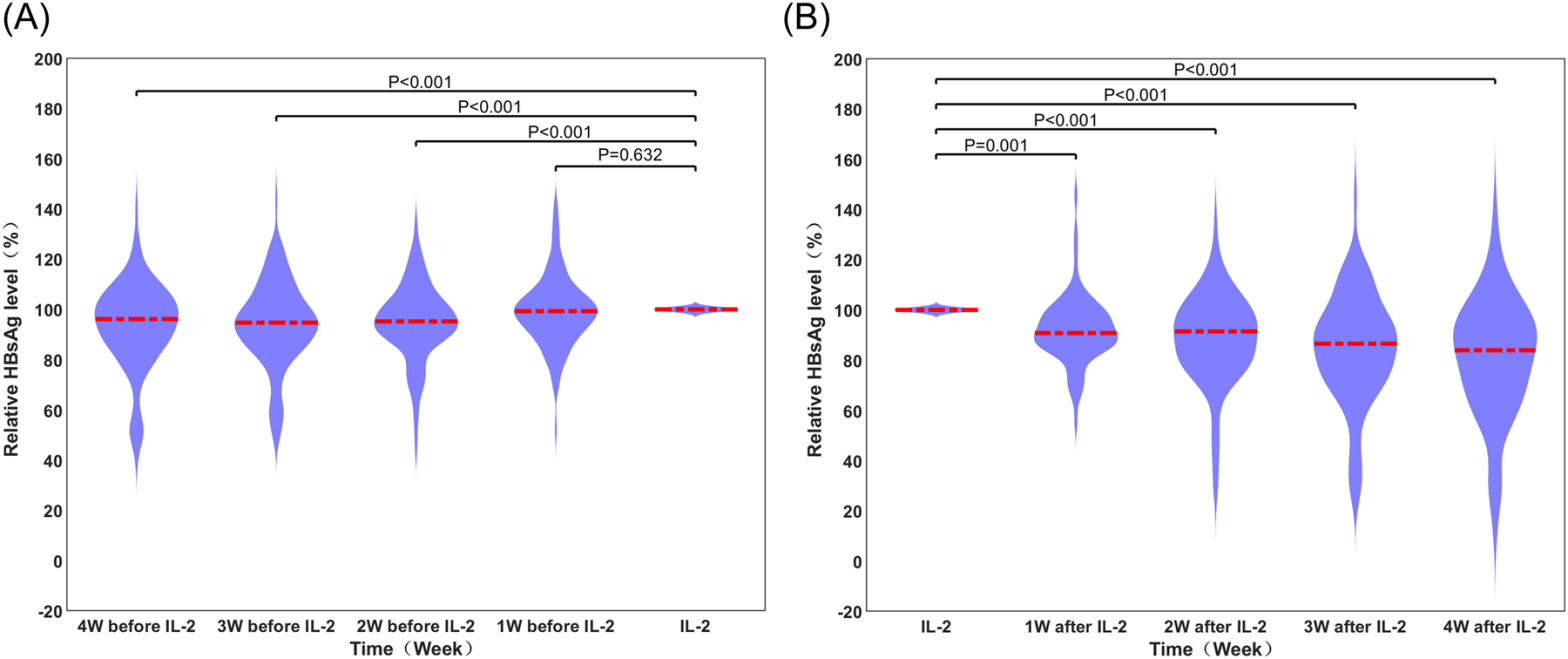
Violin plot of relative HBsAg levels before and after IL‐2 intervention. (A) Before intervention (*n* = 115); (B) after intervention (*n* = 76). The red dotted line indicates the corresponding median. HBsAg, hepatitis B surface antigen; IL‐2, interleukin‐2.

The relative HBsAg level showed an overall increasing trend for 4 consecutive weeks before IL‐2 intervention (Figure 1A). Compared with the baseline, except for the first week before intervention, the differences in the other 3 weeks were statistically significant.

Of the 115 patients, 76 received more than 4 weeks of treatment after IL‐2 intervention. Figure 1B shows that after IL‐2 intervention, the downward trend of the relative HBsAg level was significant compared with that at baseline, and the differences for all 4 weeks were statistically significant.

### 3.3 Cluster analysis of the relative change in the HBsAg level before IL‐2 intervention

**Figure 2:**
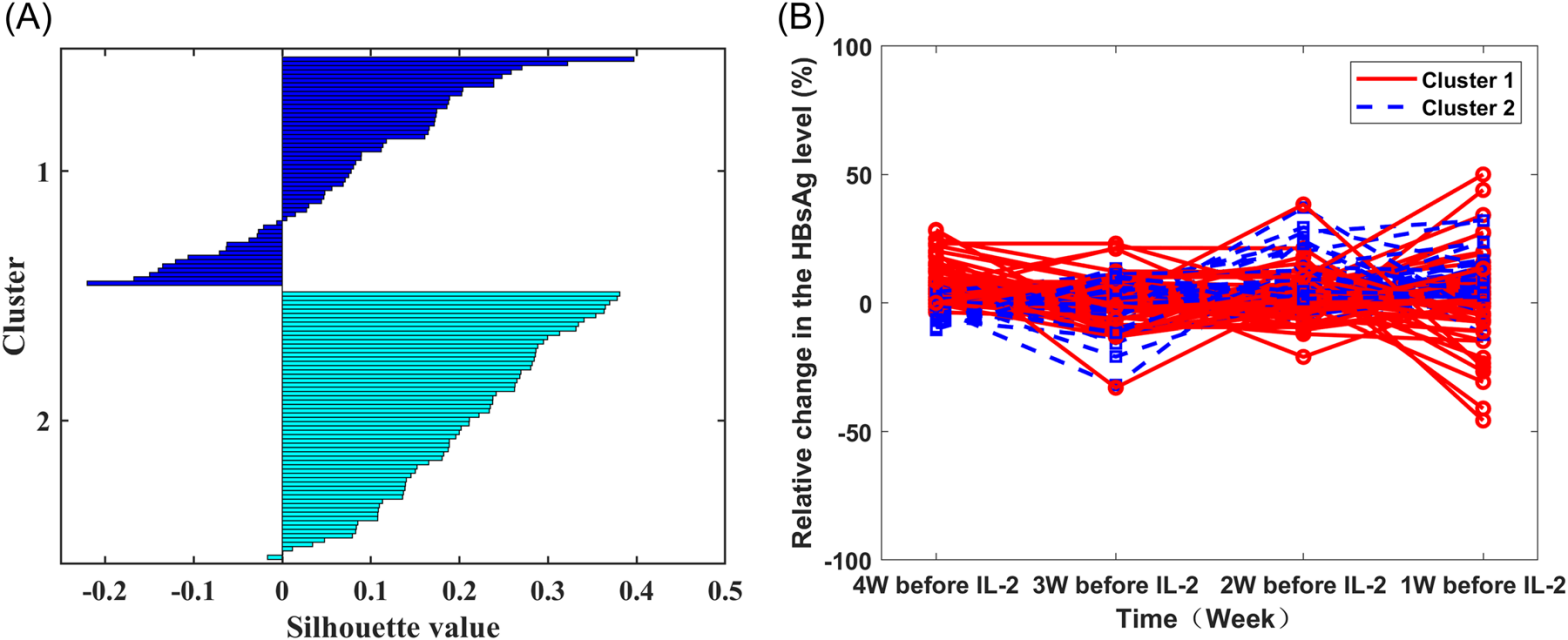
Cluster analysis results before IL‐2 intervention. (A) Silhouette value; (B) curves for the relative change in the HBsAg level in all 115 patients. HBsAg, hepatitis B surface antigen; IL‐2, interleukin‐2.

K‐medoids cluster analysis indicated that the 115 patients could be divided into two categories based on the relative change in the HBsAg level across the 4 weeks before IL‐2 intervention. The silhouette values of the cluster analysis are indicated in Figure 2A, and Figure 2B shows the relative HBsAg change level curves for all 115 patients during the 4 weeks before IL‐2 intervention, in which cluster 1 comprised 53 patients and cluster 2 comprised 62 patients.

### 3.4 Profile analysis of the relative change in the HBsAg level before and after IL‐2 intervention

**Figure 3:**
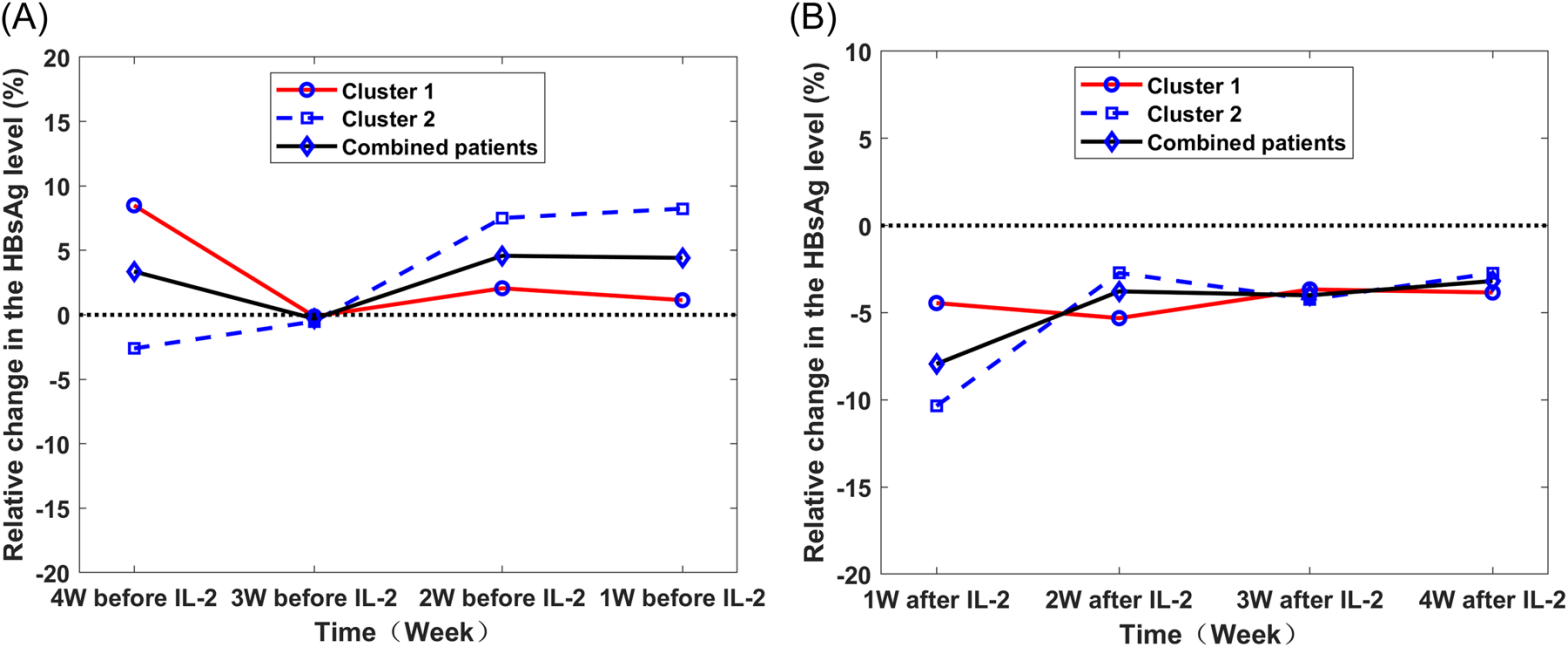
Profile analysis of the relative change in the HBsAg level before and after IL‐2 intervention. (A) Before intervention; (B) after intervention. HBsAg, hepatitis B surface antigen; IL‐2, interleukin‐2.

For the two clusters of patients, as shown in Figure 2, before IL‐2 intervention, the profile analysis results showed that cluster 1 and cluster 2 presented fluctuation platform patterns and stepwise growth patterns, respectively (Figure 3A). Most of the relative changes in the HBsAg levels were positive; that is, the Peg‐IFN response was not good. A parallel profile test found that relevant differences between the two clusters were statistically significant (*F* = 32.753, *p* < 0.001), indicating that the two types of patterns were not parallel.

For 76 patients treated for more than 4 weeks after IL‐2 intervention, the results of the clustering profile are shown in Figure 3B. Although the contours of the two categories intersected, the parallel contour test revealed that there were no significant differences between clusters (*F* = 1.773, *p* = 0.16). In addition, no statistically significant difference was found by the overlapping contour test (*F* = 0.238, *p* = 0.63). By combining the data, the horizontal contour test indicated that the relative change in the HBsAg level tended to increase gradually (*F* = 2.951, *p* = 0.04). Notably, almost all the relative changes in the HBsAg level were negative, that is, the IL‐2 intervention was effective.

### 3.5 Demographics and baseline comparison of the two categories of patients

The comparison results of the two clusters of patients are shown in Table 2. The results showed that there was a statistically significant difference in HBsAg levels (*p* < 0.001), but there was no statistically significant difference in demographics and other baseline indicators, although the patients in the stepwise growth category (cluster 2) exhibited relatively high levels of CD8+ T and NK cells and a high proportion of viremia.

**Table 2:** Comparison results of demographics and baseline characteristics between the two categories of patients

Variable	Categories of patients		Statistics	*p* Value
	Cluster 1 (*n* = 53)	Cluster 2 (*n* = 62)		
Age (year)^a^ $\bar{x}\;\pm \;\text{SD}$	42.98 ± 10.18	41.15 ± 10.87	*t* = 0.929	0.36
HBsAg (IU/ml)^b^	144.22	2188.99	*Z* = −3.939	<0.001
Median (range)	(0.10−15,187.00)	(0.85−53,110.60)		
ALT (IU/L)^b^ ^,^ ^c^	(*n* = 53)	(*n* = 57)	*Z* = −0.401	0.69
Median (range)	61	60		
	(8−420)	(13−287)		
Neutrophil level (10^9^/L)^b^ ^,^ ^c^	(*n* = 52)	(*n* = 57)	*Z* = −0.906	0.37
Median (range)	1.3	1.4		
	(0.7−4.5)	(0.6−5.2)		
Course of treatment before IL‐2 intervention (weeks)^b^	17	14	*Z* = −1.183	0.24
Median (range)	(5−125)	(5−92).		
CD8+ T cells (M/L)^b^ ^,^ ^c^	(*n* = 15)	(*n* = 34)	*Z* = −0.033	0.97
Median (range)	243.0	337.5		
	(81−1221)	(124−1062)		
NK cells (M/L)^b^ ^,^ ^c^	(*n* = 15)	(*n* = 33)	*Z* = −0.011	>0.99
Median (range)	194	204		
	(65−477)	(6−1009)		
Gender^d^, *n* (%)			*χ* ^2^ = 1.288	0.26
Male	37 (69.81%)	49 (79.03%)		
Female	16 (30.19%)	13 (20.97%)		
NAs treatment before the first visit^d^, *n* (%)			*χ* ^2^ = 0.751	0.39
Treated with NAs	30 (56.60%)	40 (64.52%)		
No NAs treatment	23 (43.40%)	22 (35.48%)		
HBeAg^d^ ^,^ ^c^, *n* (%)	(*n* = 16)	(*n* = 21)	*χ* ^2^ = 0.039	>0.99
Negative	1 (6.25%)	1 (4.76%)		
Positive	15 (93.75%)	20 (95.24%)		
HBV DNA^d^ ^,^ ^c^, *n* (%)	(*n* = 22)	(*n* = 27)	*χ* ^2^ = 0.970	0.33
Viremia	6 (27.27%)	11 (40.74%)		
Aviremia	16 (72.73%)	16 (59.26%)		
Immune stage^d^ ^,^ ^c^, *n* (%)	(*n* = 22)	(*n* = 23)	*χ* ^2^ = 0.711	
Immune‐active stage	6 (27.27%)	9 (39.13%)		0.40
Immune‐inactive stage	16 (72.73%)	14 (60.87%)		

Abbreviations: ALT, alanine transaminase; HBeAg, hepatitis B e‐antigen; HBsAg, hepatitis B surface antigen; HBV, hepatitis B virus; IL‐2, interleukin‐2; NAs, nucleos(t)ide analogues; NK, natural killer.

^a^ Independent sample *t*‐test.

^b^ Mann−Whitney *U* test.

^c^ Indicates that there are missing data for this index, and *n* in the corresponding parentheses represents the actual sample size.

^d^ The *χ* ^2^ test and Fisher's exact test were performed if the theoretical frequency was less than 5.

### 3.6 Prognostic association analysis after IL‐2 intervention

The relative HBsAg level decrease after IL‐2 intervention was compared between two categories of patients, with a value >10% as a criterion. The results showed that in the first week after IL‐2 intervention, there was a significant difference between cluster 1 and cluster 2 (32.26% [10/31] vs. 55.56% [25/45], *p* = 0.05). At subsequent time points, the difference was not statistically significant.

To explore the factors affecting the change in the level of HBsAg, we carried out stepwise regression analysis with the relative change in the HBsAg level in the first week after IL‐2 intervention as the dependent variable and demographics and baseline indicators as the explanatory variables. The results showed that age, course of treatment before IL‐2 intervention, and levels of ALT, neutrophils, CD8+ T cells, and NK cells at baseline were included in the regression equation (Table 3), which means that these indicators had statistical significance for clinical prognosis after IL‐2 intervention. Notably, the coefficient of determination (*R* ^2^) was 0.631, which indicated that 63.1% of the total variation in the relative change in the HBsAg level was related to these six indicators, and the model had good goodness of fit. Furthermore, according to the absolute values of the standardized regression coefficients, we can conclude that the influence of these indicators in descending order was as follows: CD8+ T cells, ALT, age, NK cells, neutrophils, and course of treatment before IL‐2 intervention.

**Table 3:** Parameter estimations of the regression model

Model	Unstandardized coefficients		Standardized coefficients	*t*	*p* Value
	*B*	Standard error			
Constant	2.981	10.111		0.295	0.77
Age	−0.355	0.163	−0.307	−2.181	0.04
Course of treatment before IL‐2 intervention	−0.395	0.175	−0.302	−2.256	0.03
Baseline ALT	−0.065	0.023	−0.348	−2.810	0.009
Baseline neutrophils	5.012	2.113	0.304	2.372	0.03
Baseline CD8+ T cells	0.024	0.008	0.427	2.975	0.006
Baseline NK cells	−0.020	0.009	−0.306	−2.138	0.04

*Note*: *F* = 7.111, *p* < 0.001; *R* ^2^ = 0.631.

Abbreviations: ALT, alanine transaminase; IL‐2, interleukin‐2; NK, natural killer.

## 4 DISCUSSION

Relevant in vitro studies have found that interferon alpha 2a combined with IL‐2 can significantly improve the inhibitory effect on HepG2.2.15 hepatoma cells.^15^ A clinical study also showed that for entecavir‐suppressed CHB patients, especially those with low baseline HBsAg, the combination of IFN with other immunomodulators (such as IL‐2 and therapeutic vaccines) may increase HBsAg loss.^5^ In this study, we found that in the bottleneck stage of interferon treatment, that is, the stage wherein the HBsAg level does not drop but continues to rise, using IL‐2 as an adjuvant therapy can significantly improve the level of the Peg‐IFN response, resulting in a significant decrease in HBsAg.

Based on 4 consecutive weeks of indicators for the relative change in the HBsAg level, two kinetic patterns were identified: a fluctuation platform pattern (cluster 1) and a stepwise growth pattern (cluster 2). According to existing clinical data, we also found that although the overall clinical prognosis showed a trend of improvement after IL‐2 intervention in both clusters of patients, the difference in prognostic benefits was significant only after 1 week of IL‐2 intervention between the clusters.

To optimize the response to Peg‐IFN after IL‐2 intervention, according to stepwise regression analysis, we found six baseline indicators that may be related to the response, namely, age, course of treatment before IL‐2 intervention, and levels of ALT and NK cells were associated with a better response, but the opposite was true for the levels of neutrophils and CD8+ T cells. However, gender, NAs treatment before the first visit, and other baseline variables were statistically insignificant. This may be related to the sample of this study. In the course of treatment before IL‐2 intervention, since longer Peg‐IFN treatment leads to more serious depletion of CD8+ T cells,^16^, ^17^ IL‐2 intervention at this time can effectively improve the state of immune depletion, so the response is better. In addition, continuous Peg‐IFN treatment usually leads to a decline in NK cells to some extent. The less the decline is, the more residual NK cells and the stronger the response may be. As a result, a better response can be induced after Il‐2 intervention due to the effective stimulation of NK cells.^18^ Since the level of ALT is closely and positively correlated with the degree of immune activity,^19^ the characteristics of the dominant population for interferon therapy include high baseline ALT levels.^20^ Therefore, expert consensus suggests that PEG‐IFN treatment should be given priority to high‐baseline ALT.^21^ In fact, a meta‐analysis for CHB in children found that children with high ALT levels had a better response to interferon‐α therapy,^22^ and a recent study also found that the clearance of both serum HBV markers and intrahepatic HBV DNA was associated with serum ALT elevations and liver infiltrations by CD4 and CD8 T cells.^23^ As a result, after excluding the increase in ALT caused by drugs, alcohol, and other factors, a higher ALT level indicates more active viral clearance, which should be the case for both interferon therapy and IL‐2 intervention. The lower the baseline CD8+ T cells, the better the response. This may be related to the recovery of immune function after immune depletion, but further verification is needed. It has been reported that younger people have a better response,^21^ but surprisingly, there was an opposite relationship in our data set. The reason may be associated with the synergistic effect between age and other indicators, which is worth expanding the sample size for further hierarchical verification.

Recently, a study found that during early peginterferon alpha‐2a treatment, dynamic changes in cytokine profiles and virological markers were associated with HBsAg loss in HBeAg‐positive CHB patients, but their detection time points were few.^24^ Except for the baseline, the indicators were detected only at the 12th and 24th weeks. In addition, for patients with CHB undergoing IFN‐α‐based therapies, the study found that sequential IL‐2 administration can rescue effective immune function, which showed efficacy in rescuing immune function in nonresponder patients with refractory CHB.^25^ Although there is no unified method to address the bottleneck period in the process of interferon treatment of CHB, front‐line clinicians have been constantly exploring more effective means to improve HBsAg clearance. This study reflects the effectiveness of IL‐2 intervention through intensive detection of HBsAg quantification (once a week), which is different from other studies.

However, the present study has some limitations that should be addressed in future studies. First, as a retrospective study, this study involved only single‐center patient data and was limited by patient compliance during treatment. The sample size was relatively small, and selection bias was inevitable; thus, the sample size should be expanded to further strengthen the conclusion. Second, as a pragmatic clinical study, due to the limitations of hospital testing conditions and clinical treatment practice, many meaningful indicators could not be assessed and discussed. For example, according to the role of IL‐2,^26^ the influence of the internal concentration of IL‐2 before treatment was not discussed in this study. Third, the patients were all from Zhejiang Province, China, which limits the extrapolation of the conclusions to other regions and races.

## 5 CONCLUSION

For CHB patients who have received continuous Peg‐IFN‐α treatment, IL‐2 intervention should be given as early as possible when the HBsAg level has not decreased for 4 consecutive weeks or a fluctuation platform pattern is observed. After the intervention, a downward relative change in the level of HBsAg can be maintained over 4 weeks. CD8+ T cells, ALT, NK cells, and neutrophils are baseline indicators closely related to the prognosis of the intervention.

## AUTHOR CONTRIBUTIONS

**Wencai Qi**: data curation; formal analysis; software; visualization; writing – original draft. **Yuming Wang**: conceptualization; supervision. **Guangyu Huang**: conceptualization; data curation; investigation; methodology; project administration; resources; writing – original draft; writing – review & editing. **Kaifa Wang**: conceptualization; data curation; formal analysis; funding acquisition; methodology; project administration; resources; software; visualization; writing – original draft; writing – review & editing.

## CONFLICT OF INTEREST

The authors declare no conflict of interest.

## ETHICS STATEMENT

Participant consent is not applicable as the data was required only from the Patient's Medical record. Whereas the study got an exemption from the Ethical Review Committee of the Fourth Affiliated Hospital Zhejiang University School of Medicine. All methods were performed in accordance with the relevant guidelines and regulations. Consent was obtained for all patient records and personally identifiable data.

## TRANSPARENCY STATEMENT

The lead author Guangyu Huang, Kaifa Wang affirms that this manuscript is an honest, accurate, and transparent account of the study being reported; that no important aspects of the study have been omitted; and that any discrepancies from the study as planned (and, if relevant, registered) have been explained.

## Supporting information

Supplementary information.

## Data Availability

All authors have read and approved the final version of the manuscript. Kaifa Wang and Guangyu Huang had full access to all of the data in this study and takes complete responsibility for the integrity of the data and the accuracy of the data analysis.
