# Supplementary material for: Interleukin‐2 promotes pegylated interferon alpha for hepatitis B surface antigen loss: A retrospective pragmatic clinical study at the Fourth Affiliated Hospital of Zhejiang University Medical College
Source: Health Sci Rep. 2022 Nov 14;5(6):e932. doi: 10.1002/hsr2.932 (PMC9662690; doi:10.1002/hsr2.932)
Supplement: Supplementary file 1 — Supplementary information. [file HSR2-5-e932-s001.docx]

**Supporting information**

The selection and implementation of statistical methods in this study are summarized as follows:

For categorical data, frequencies or percentages are presented as needed. Chi-square test is performed by SPSS. If the total sample size is less than 40 or the theoretical frequency is less than 5, Fisher's exact test is performed, and the exact corrected probability value P is reported.

For quantitative data, normal fitting test is first performed by Shapiro-Wilk test. If the data is normally distributed, it is expressed as the mean ± standard deviation (SD), the independent sample *t*-test is performed to test whether the difference between the mean values of the two groups is statistically significant. If the data does not follow the normal distribution, it is expressed as the median with a corresponding range, Mann-Whitney U test is performed to test whether the mean values of the two groups are significantly different. Note that the relative HBsAg levels at different time points are repeated measurement data. Friedman rank test is performed to test the difference at different time points. These statistics, including profile analysis and stepwise regression analysis (backward), are analyzed in SPSS.

K-medoids cluster analysis is an improved method of K-means. K-means takes the average value of all objects in this class as particle, and then uses the partition method to split the original dataset into K groups based on the distance between objects and the particle, so K-means is more sensitive to outliers. However, K-medoids selects the object with the smallest sum of distances from all other objects in the current class as particle, so it is not sensitive to outliers, though it has a large amount of computation and is generally suitable for small data. Due to the small amount of data in this study (only 115 patients' clinical data) and the existence of outliers, in order to avoid the distortion of clustering results caused by dirty data, we choose K-medoids to cluster patients. Specifically, we use *pam* function in the *cluster* package to implement clustering algorithm in R software.
